# Nasal TSLP and periostin in infants with severe bronchiolitis and risk of asthma at 4 years of age

**DOI:** 10.1186/s12931-023-02323-7

**Published:** 2023-01-24

**Authors:** Maria Luz Garcia-Garcia, Beatriz Sastre, Maria Arroyas, Maite Beato, Patricia Alonso, José Manuel Rodrigo-Muñoz, Victoria Del Pozo, Inmaculada Casas, Cristina Calvo

**Affiliations:** 1 Pediatrics Department, Hospital Universitario Severo Ochoa, Avenida Orellana s/n, 28911 Leganés, Madrid Spain; 2 Fundación para la Investigación Biomédica, Hospital Universitario Puerta de Hierro, Majadahonda, Spain; 3 CIBER de Enfermedades Infecciosas (CIBERINFEC), Madrid, Spain; 4 Translational Research Network in Pediatric Infectious Diseases (RITIP), Madrid, Spain; 5 Department of Immunology, IIS-Fundación Jiménez Díaz, Madrid, Spain; 6 CIBER de Enfermedades Respiratorias (CIBERES), Madrid, Spain; 7 Respiratory Virus and Influenza Unit, National Microbiology Centre (ISCIII), Madrid, Spain; 8 Pediatric Infectious Diseases Department, Hospital Universitario La Paz, Madrid, Spain; 9 Fundación IdiPaz, Madrid, Spain; 10 TEDDY Network (European Network of Excellence for Pediatric Clinical Research), Madrid, Spain

**Keywords:** Bronchiolitis, Asthma, Recurrent wheezing, Periostin, Thymic stromal lymphopoietin (TSLP), Respiratory syncytial virus (RSV), Rhinovirus (HRV)

## Abstract

**Background:**

Severe bronchiolitis is often associated with subsequent respiratory morbidity, mainly recurrent wheezing and asthma. However, the underlying immune mechanisms remain unclear. The main goal of this study was to investigate the association of nasal detection of periostin and thymic stromal lymphopoietin (TSLP) during severe bronchiolitis with the development of asthma at 4 years of age.

**Methods:**

Observational, longitudinal, post-bronchiolitis, hospital-based, follow-up study. Children hospitalized for bronchiolitis between October/2013 and July/2017, currently aged 4 years, included in a previous study to investigate the nasal airway secretion of TSLP and periostin during bronchiolitis, were included. Parents were contacted by telephone, and were invited to a clinical interview based on a structured questionnaire to obtain information on the respiratory evolution. The ISAAC questionnaire for asthma symptoms for 6–7-year-old children, was also employed.

**Results:**

A total of 248 children were included (median age 4.4 years). The mean age at admission for bronchiolitis was 3.1 (IQR: 1.5–6.5) months. Overall, 21% had ever been diagnosed with asthma and 37% had wheezed in the last 12 months. Measurable nasal TSLP was detected at admission in 27(11%) cases and periostin in 157(63%). The detection of nasal TSLP was associated with the subsequent prescription of maintenance asthma treatment (p = 0.04), montelukast (p = 0.01), and the combination montelukast/inhaled glucocorticosteroids (p = 0.03). Admissions for asthma tended to be more frequent in children with TSLP detection (p = 0.07). In the multivariate analysis, adjusting for potential confounders, the detection of TSLP remained independently associated with chronic asthma treatment prescription (aOR:2.724; CI 1.051–7.063, p:0.04) and with current asthma (aOR:3.41; CI 1.20–9.66, p:0.02).

Nasal detection of periostin was associated with lower frequency of ever use of short-acting beta2-agonists (SABA) (p = 0.04), lower prevalence of current asthma (p = 0.02), less prescription of maintenance asthma treatment in the past 12 months (p = 0.02, respectively). In the multivariate analysis, periostin was associated with lower risk of asthma at 4 years, independently of the atopic status (aOR:0.511 CI 95% 0.284–0.918, p:0.025).

**Conclusions:**

Our results show a positive correlation between nasal TSLP detection in severe bronchiolitis and the presence of current asthma, prescription of asthma maintenance treatment and respiratory admissions up to the age of 4 years. By contrast, we found a protective association between nasal periostin detection and current asthma at 4 years, ever diagnosis of asthma, maintenance asthma treatment prescription, and respiratory admissions.

## Background

Acute bronchiolitis is a common lower respiratory tract infection in infants, often caused by respiratory viruses, and accounts for up to 15–17% of all hospitalizations in infants under 2 years [1]. Respiratory syncytial virus (RSV) is the most common cause of bronchiolitis, although other viruses, mainly rhinovirus (HRV) are also frequently identified in these infants. RSV and HRV account for 60–80% of bronchiolitis in infants [2].

It is well known that severe bronchiolitis is often associated with subsequent respiratory morbidity and up to 30–40% of infants hospitalized for bronchiolitis will develop recurrent wheezing or asthma later in life [3–6]. However, not all children hospitalized with bronchiolitis will develop asthma. The mechanisms underlying asthma following bronchiolitis hospitalization are complex and immune responses to respiratory viruses may underlie both, bronchiolitis severity and long-term sequela such as asthma [7]. However, the underlying immune mechanisms of chronic respiratory illness development after acute bronchiolitis remain unclear. Several cytokines have been involved in Th2-asthma, among them, the alarmins, initiators of T2 inflammation: IL-33, IL-25, and thymic stromal lymphopoietin (TSLP). Alarmins promote the expression of IL-4, IL-5, and IL-13 and increase the levels of periostin [8].

TSLP, an epithelial cell-derived cytokine, is synthesized in response to various stimuli such as RSV infections and is considered a master regulator of type 2 immune response in the respiratory tract, that links the innate and adaptive immune responses by activating innate lymphoid cells (ILC2), as well as inducing Th2-type T cell differentiation [9, 10].

Periostin is a matricellular protein produced in response to inflammatory stimuli mediated by IL-4, IL-5, and IL-3 by many cells, including epithelial cells and fibroblasts. There is evidence that periostin modulates upper respiratory tract inflammation and remodeling, can induce the differentiation of fibroblasts into myofibroblasts and increase fibrosis, can influence epithelial remodeling, and can change the underlying matrix by modifying the deposition of collagen fibrils [11].

There is growing evidence that TSLP and periostin are elicited in the upper airways of infants with RSV and HRV bronchiolitis, and increased TSLP levels are related to more severe disease and intensive care unit (ICU) admission [12]. Lee et al. [13] reported that viral antigen recognition triggers a signalling cascade that results in TSLP production and strong T2 response that seems to play a key role in the pathogenesis of asthma. Indeed, the Th1/Th2 imbalance has been proposed as a key event in the inflammatory process after severe bronchiolitis that could predispose to recurrent wheezing and asthma [14]. It has been also proposed that several T2 cytokines such as TSLP or periostin could be used as prognosis biomarkers for the development of asthma [15].

The main goal of this study was to evaluate whether there is some association between the nasal detection and the levels of TSLP and periostin, in infants admitted for bronchiolitis, and the subsequent development of recurrent wheezing and asthma at 4 years of age. Other secondary outcomes, also related with the association of nasal TSLP and periostin with the long-term respiratory morbidity, were evaluated, mainly need of chronic asthma treatment and respiratory admissions during the follow-up period.

## Methods

### Study design

This was an observational, longitudinal, post-bronchiolitis, hospital-based, follow-up study, which is a part of an ongoing prospective investigation of respiratory tract infections in children, approved by the Medical Ethics Committee. Written informed consent was obtained from all the parents/caregivers after a full explanation of the study protocol. All methods were carried out in accordance with relevant guidelines and regulations.

### Clinical assessment

Children hospitalized for their first episode of acute bronchiolitis in the Severo Ochoa University Hospital (Spain), between October 2013 and July 2017, currently aged 4 years, included in a previous study to investigate whether infants exhibit enhanced nasal airway secretion of TSLP, IL-33, and periostin during natural respiratory viral bronchiolitis, were included [12]. Parents were contacted by telephone, and were invited to a clinical interview based on a structured questionnaire to obtain information on wheezing episodes; bronchodilator and oral corticosteroid prescription; related hospital admissions; chronic asthma treatment; physician-diagnosed atopic dermatitis; allergic rhinitis; food allergy; pet contacts; daycare attendance; parental smoking habits; allergy; eczema and asthma in first order family members diagnosed by a medical doctor. The ISAAC questionnaire for asthma symptoms for 6–7-year-old children, previously validated and translated to Spanish was also employed [16]. To minimize recall bias, data reported by parents were confirmed by reviewing electronic medical records from both, hospital and primary care.

*Current asthma* prevalence was estimated by the proportion of patients who responded positively to question number 2 of the ISAAC questionnaire (wheezing or whistling in the chest in the last 12 months), the one which has demonstrated the greatest correlation with current asthma prevalence in validation studies [16]. The prescription of chronic asthma treatment and the need for respiratory admissions during the follow-up were considered and evaluated as indicators of asthma severity.

*Recurrent wheezing* was defined as the presence of wheezing diagnosed by a doctor in the first 4 years of life [17].

The classic criteria, an initial episode of acute onset expiratory dyspnoea with previous signs of viral respiratory infection—whether this was associated with respiratory distress or pneumonia—were applied in diagnosing *bronchiolitis* [18].

### Virus detection

Two nasopharyngeal samples (NPA) were obtained at admission for bronchiolitis by a standard, routine technique, consisting of gently washing the nasal cavity with 1 ml of phosphate buffered saline in each nostril and collection into a standard mucus extractor. The samples were refrigerated at 4 °C until being processed within 24 h of collection. One of the two samples was processed in the Respiratory Virus and Influenza Unit at the National Microbiology Centre (ISCIII, Madrid, Spain). Detection of respiratory virus was performed by 3 independent multiplex reverse transcription-polymerase chain reaction (RT-PCR) assays. The first assay detected Influenza A, B, and C viruses; the second was used to detect parainfluenza viruses (PIV) 1 to 4, HRV, and enteroviruses; and the third assay detected the presence of RSV types A and B, human metapneumovirus (HMPV), human bocavirus (HBoV), and human adenoviruses (ADV). These 3 assays were real-time multiplex RT-PCRs and used the SuperScript™ III Platinum^®^ One-Step Quantitative RT-PCR System (Invitrogen). The other sample was used for immunological testing at the Immunology Department of IIS-Fundación Jiménez Díaz as described below.

A recent study by Lopez-Guisa et al. [19] demonstrated a good correlation between bronchial and nasal epithelial expression of pro-remodelling factors. NPA is a non-invasive method, especially useful in infants and young children.

### Detection of cytokines and proteins in nasal secretions

#### Nasopharyngeal aspirate processing

Previous to NPA filtrations with a 40-µm nylon filter, NPAs were centrifuged and cellular pellet and supernatant were obtained. Supernatants were directly frozen at – 80 °C.

#### Immunological analyses in nasopharyngeal aspirate

In NPA supernatant TSLP and periostin were analysed by ELISA Kit (R&D Systems, Abingdon, UK), according to the manufacturer’s instructions using provided standards and quality controls. The intra-assay and inter-assay coefficients of variation were: TSLP: 8.2% and 7.47%, respectively, and periostin: 2.19% and 9.99%, respectively. The lower detection limit of these assays was 32.5 pg/ml for TSLP and 62.5 pg/ml for periostin.

### Statistical analysis

Values were expressed as percentages for discrete variables, or as mean and standard deviation or median and interquartile range for continuous variables. Comparisons used either X^2^ or Fisher exact test (2-tailed) for categorical variables and Student *T*-test, Mann–Whitney *U* test, Kruskal–Wallis test, and analysis of variance (ANOVA) for continuous variables. To control for potentially confounding variables (maternal/paternal/siblings’ asthma and atopy, atopic dermatitis, prematurity, viral identification during acute bronchiolitis, and cigarette smoke exposure) and to examine the independent association between nasal TSLP and periostin and the likelihood of developing asthma, a backward stepwise binomial logistic regression model was built. All the variables with p-value < 0.1 were introduced in the multi-variate analysis. Adjusted odds ratios (OR) with 95% confidence intervals were calculated. A probability of < 0.05 was considered statistically significant. All analyses were performed using the Statistical Package for the Social Sciences (SPSS), Version 23.0.

## Results

The study population consisted of 323 patients admitted for bronchiolitis and current aged 4 years. Of them, 248 could be located and accepted to participate by telephone, answering the clinical questionnaires. The main reason for the drop-out was a change in their telephone number. Children who lost follow-up did not differ significantly from others regarding initial hospitalization, gender, type of virus, prematurity, and age at inclusion. The mean age at admission for bronchiolitis was 3.1 (IQR:1.5–6.5) months. The median age at inclusion in the follow-up study was 4.4 (IQR 3.7–5.1) years and 146 (59%) of them were males (Table 1).

**Table 1:** Background and clinical characteristics at admission of enrolled infants with bronchiolitis (N = 248)

Clinical characteristic	
Current age^a^ (years)	4.4 (3.7–5.1)
Age at admission^a^ (months)	3.1 (1.5–6.51)
Male (N, %)	159 (59%)
Temperature ≥ 38 °C (N, %)	103 (38.6%)
SatO2 < 95% (N, %)	199 (74.5%)
Length of hospital stay^a^ (days)	4.5 (3.2)
Infiltrate/atelectasis (N, %)	42 (15.7%)
ICU admission (N, %)	10 (3.7%)
Antibiotic treatment (N, %)	30 (11.5%)
Leucocytes^a^ (cells/mcl)	12,615 (8950–15,755)
Reactive C protein^a^ (mg/L)	10.5 (4–29)
Prematurity (N, %)	29 (11.6%)
Breastfeeding (N, %)	229 (87.4%)
Day care centre attendance (N, %)	20 (7.5%)
Siblings < 5 years (N, %)	91 (36%)
Atopic dermatitis (N, %)	112 (43%)
Asthma (N, %)	
Mother	15 (6%)
Father	18 (7.4%)
Siblings	34 (15.5%)
Atopy (N, %)	
Mother	33 (13.6%)
Father	40 (16.5%)
Siblings	20 (9.6%)
Smoking (N, %)	
Mother	26 (15%)
Father	51 (30%)
Maternal smoking in pregnancy (N, %)	29 (13%)

^a^Median (interquartile range)

### Clinical characteristics at admission

Personal and family backgrounds, as well as clinical characteristics during admission for bronchiolitis, are shown in Table 1. A total of 215 (87%) cases had a positive respiratory viral identification. Of them, the most frequent were RSV (167/78%), followed by HRV (65/30%), PIV (17/8%), ADV (14/6.5%), HMPV (13/6%) and HBoV (10/4.6%). Simultaneous respiratory viral detection occurred in 64 (26%) cases, and the most common combination was RSV/HRV in 40 (62.5%) cases.

Measurable nasal levels of TSLP were detected at admission for bronchiolitis in 27 (11%) cases and periostin in 156 (63%).

### Medium-term respiratory evolution

After admission for bronchiolitis, 182 (73%) children reported recurrent wheezing in the follow-up. The current prevalence of asthma, according to the affirmative response to question number 2 of the ISAAC questionnaire, was 37% (Table 2).

**Table 2:** Respiratory evolution at 4 years of age after admission for bronchiolitis (N = 248)

Clinical features	(N, %)
Recurrent wheezing	181 (73%)
Recurrent wheezing in the last 12 months	93 (37%)
Emergency care attendance for wheezing	126 (51%)
Emergency care attendance for wheezing in the last 12 months	49 (20%)
Admission for wheezing episode	49 (20%)
Admission for wheezing episode in the last 12 months	12 (5%)
Acute oral corticosteroid treatment	112 (45%)
Acute oral corticosteroid treatment in the last 12 months	43 (17%)
Chronic asthma treatment prescription	94 (38%)
Chronic asthma treatment prescription in the last 12 months	35 (14%)
Montelukast treatment	38 (15%)
Inhaled glucocorticoid treatment	56 (22.5%)
Montelukast + Inhaled glucocorticoid treatment	28 (11%)
Inhaled glucocorticoid + long beta 2 agonist treatment	4 (1.6%)
ISAAC—asthma questionnaire	
Question 1. Ever wheezing in the chest at any time in the past	197(79%)
Question 2. Wheezing in the chest in the last 12 months (“Current asthma”)	92 (37%)
Question 5. In the last 12 months, wheezing severe enough to limit speech	10 (4%)
Question 6. Ever asthma	53(21%)
Question 7. In the last 12 months, chest sounded wheezy during or after exercise	27 (11%)
Question 8. In the last 12 months, dry cough at night, apart from a cough associated with a cold or a chest infection	35 (14%)

### Relationship between nasal levels of TSLP and periostin and respiratory morbidity at 4 years of age

#### TSLP

Nasal TSLP was detected in 27 patients, most of them with positive viral detection (85%). Infants with RSV + RV coinfection were 2.7 times more likely to have detectable nasal levels of TSLP than single-RSV ones (p = 0.04). However, no difference was found between RSV + RV coinfections and single-RV infections or among the other respiratory viruses. Other variables also associated with TSLP detection were intensive care unit (ICU) admission (p = 0.02) and siblings with asthma/atopy (p = 0.002) (Table 3).

**Table 3:** Clinical characteristics and respiratory evolution of children with thymic stromal lymphopoietin (TSLP) measured at admission for bronchiolitis according todetectable vs. non-detectable TSLP

	Detectable TSLP (N = 27) N(%)	Non-detectable TSLP (N = 195) N(%)	p-value
Male sex	16(59%)	111(57%)	0.818
Age at admission < 6 months	19(91%)	107(72%)	**0.06**
ICU admission	3(12%)	5(2.5%)	**0.02**
Premature birth	3(11.5%)	21(12%)	0.992
Atopic dermatitis	13(48%)	81(42%)	0.529
Siblings with asthma	10(40%)	24(15%)	**0.002**
Passive tobacco exposure	10(40%)	50(30%)	0.197
Positive viral detection	23(85%)	168(86%)	0.620
Viral coinfection	10(43%)	45(26%)	0.09
RSV infection	20(83%)	133(71%)	0.220
RV infection	9(39%)	45(27%)	0.211
RSV + RV coinfection vs. RSV single infection	8(42%)	28(21%)	**0.04**
RSV + RV coinfection vs. RV single infection	8(90%)	28(62%)	0.121
Recurrent wheezing	20(74%)	141(72%)	0.847
Hospitalization for asthma	9(33%)	36(19%)	0.08
Asthma maintenance treatment	15(56%)	68(35%)	**0.04**
Inhaled corticosteroids treatment	10(38.5%)	41(22%)	**0.07**
Montelukast	9(35%)	27(15%)	**0.01**
Inhaled corticosteroids + montelukast	6(23%)	17(9%)	**0.03**
Recurrent wheezing	20(74%)	141(72%)	0.847
Admission for asthma	9(33%)	36(19%)	**0.07**
Ever wheezing	22(85%)	150(79%)	0.501
Wheezing in the past 12 months	6(23%)	72(38%)	0.140
Ever asthma	6(23%)	43(23%)	0.878
Wheezing after exercise	3(11%)	21(11%)	0.927
Dry cough at night	3(11%)	25(13%)	0.832

Quantitatively, higher concentrations of TSLP were also detected in infants with RSV + RV coinfection (p = 0.01), ICU admission (p = 0.01), those with siblings with asthma/atopy (p = 0.004), and first-degree family history of atopy (p < 0.001). Although no significant differences were observed regarding mean age at admission, infants under 6 months were more likely to have detectable levels of TSLP (p = 0.06) than those older that age.

Regarding the medium-term respiratory morbidity, the detection of nasal TSLP was significantly associated with the prescription of maintenance asthma treatment (p = 0.04), prescription of montelukast (p = 0.01), and the combination montelukast plus inhaled glucocorticosteroids (IGC) (p = 0.03). IGC treatment (p = 0.07) and any admission for asthma (p = 0.07), tended to be more frequent in children with TSLP detection, although without reaching statistical significance (Table 3). In the multivariate analysis, adjusting for potential confounders (age at admission, RSV/HRV coinfection, siblings with asthma, maternal asthma/atopy, atopic dermatitis, and ICU admission), the detection of TSLP was independently associated with current asthma (aOR:3.41; CI 1.20–9.66, p: 0.02), prescription of chronic asthma treatment (aOR: 2.724; CI 1.051–7.063, p: 0.04), and maternal asthma (aOR: 2.884; CI 1.199–6.937, p: 0.02) (Table 4).

**Table 4:** Medium-term (4 years of age) respiratory variables independently associated with nasal TSLP and periostin detection at admission for bronchiolitis

TSLP nasal detection	p-value	Adjusted Odds Ratio	Confidence interval 95%
Current asthma at 4 years of age (wheezing in the past 12 months)	0.02	3.41	1.20–9.66
Chronic asthma treatment	0.04	2.72	1.04–7.06
Maternal asthma	0.02	2.88	1.19–6.93

Periostin nasal detection	p-value	Adjusted Odds Ratio	Confidence interval 95%
Current asthma at 4 years of age (wheezing in the past 12 months)	0.02	0.51	0.28–0.92

**Fig. 1:**
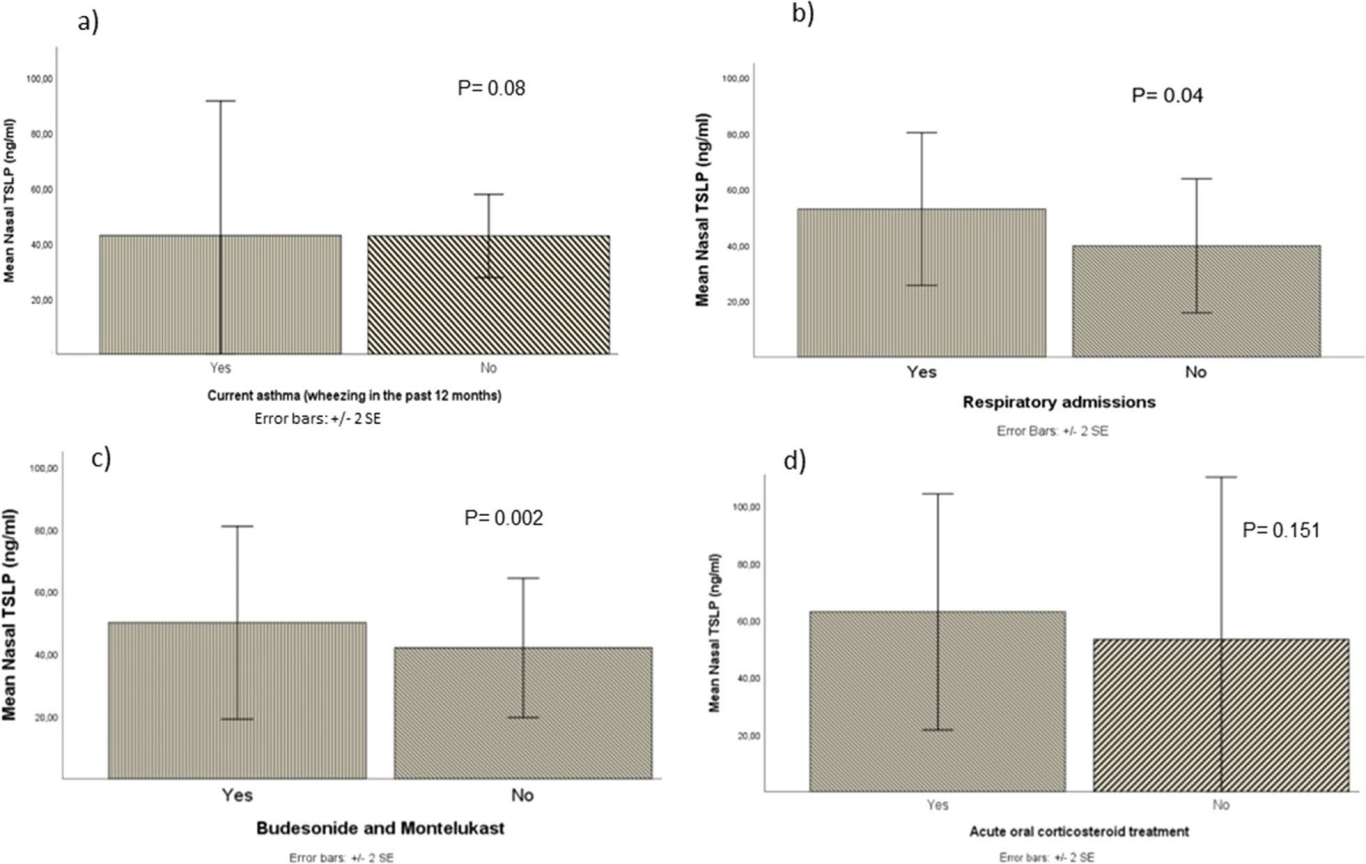
Comparison of mean nasal levels and standard error (SD) of TSLP in children with **a** Current asthma; **b** Respiratory admissions, **c** Budesonide and Montelukast treatment, **d** Acute oral corticosteroid treatment

Quantitatively, higher levels of nasal TSLP were detected in infants who subsequently required respiratory admissions (p = 0.04), were prescribed montelukast (p = 0.04), or the combination montelukast/IGC (p = 0.002). Children with current asthma tended to have higher levels of nasal TSLP at admission, although the difference did not reach statistical significance (p = 0.08). No differences regarding bronchodilator treatment or oral corticosteroid prescription were observed (Fig. 1).

#### Periostin

Periostin was detected in 156 cases, more often in infants with a maternal history of atopy (p = 0.015) and in those with RSV-bronchiolitis (p = 0.06) (Table 5).

**Fig. 2:**
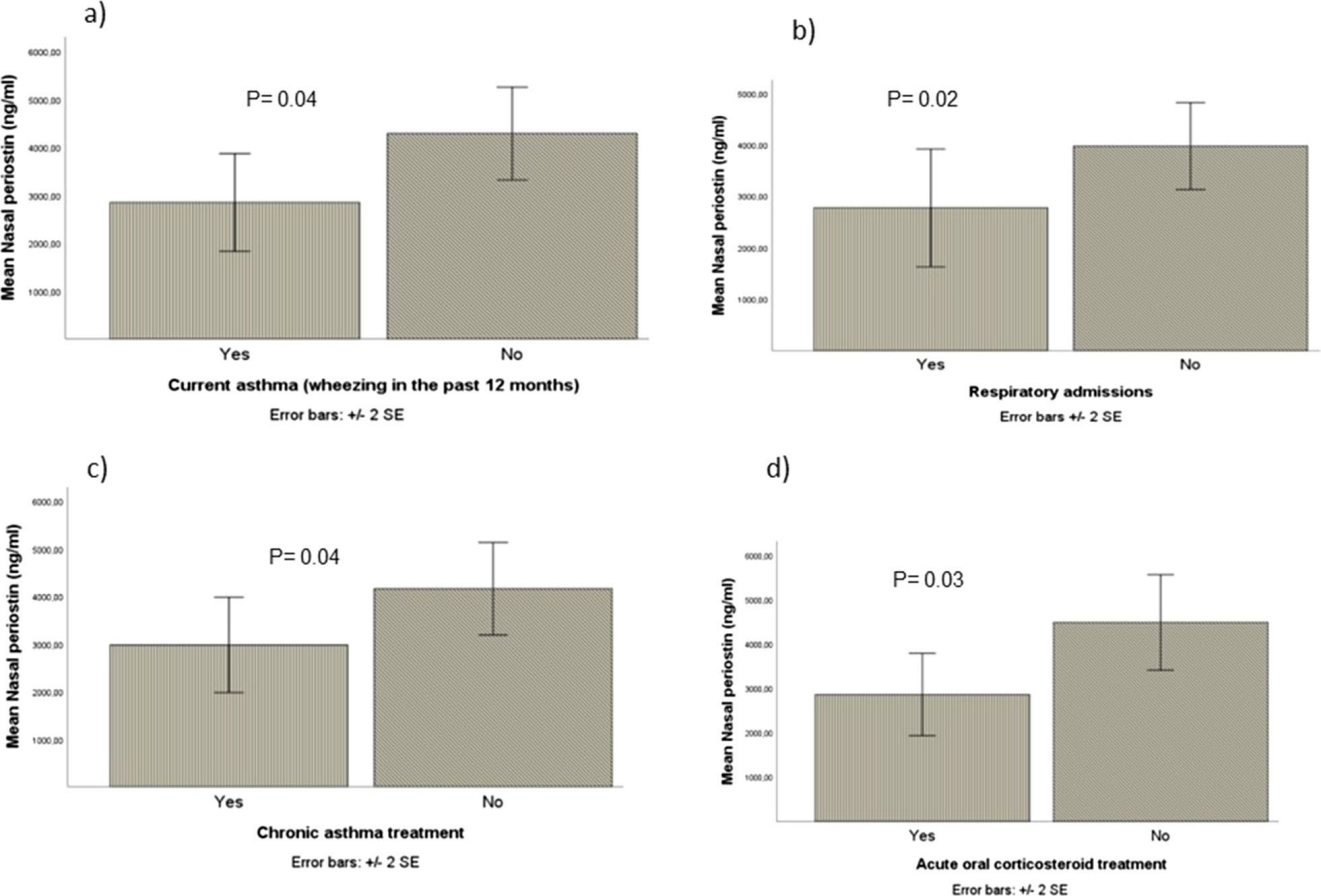
Comparison of mean nasal levels and standard error (SD) of periostin (ng/ml) in children with, **a** Current asthma; **b** Respiratory admissions, **c** Chronic asthma treatment, **d** Acute oral corticosteroid treatment

Concerning the medium-term respiratory evolution, the levels of nasal periostin tended to be higher in those infants who did not develop recurrent wheezing (p = 0.06), never needed respiratory admissions (p = 0.02), never received inhaled bronchodilators (p = 0.02) or oral corticosteroids (p = 0.03), were not prescribed chronic maintenance treatment for asthma (p = 0.002) and did not report current asthma (p = 0.004) (Fig. 2).

Children with detection of nasal periostin at admission for bronchiolitis, showed lower frequency of ever use of short-acting beta2-agonists (SABA) (p = 0.04), lower prevalence of current asthma (p = 0.02), and less prescription of SABA and maintenance asthma treatment in the past 12 months (p = 0.002 and p = 0.02, respectively) (Table 5).

**Table 5:** Clinical characteristics and respiratory evolution of children with periostin measured at admission for bronchiolitis according to detectable vs. non-detectable periostin

	Detectable periostin (N = 156) N (%)	Non-detectable periostin (N = 87) N (%)	p-value
Male sex	92(59%)	49(56%)	0.688
Age at admission < 6 months	113(72%)	64(75%)	0.578
ICU admission	25(16%)	13(15%)	0.824
Premature birth	18(12%)	8(10%)	0.671
Maternal atopy	26(18%)	5(6%)	**0.015**
Passive tobacco exposure	46(34%)	20(29%)	0.444
Positive viral detection	129(90%)	69(85%)	0.259
Viral coinfection	42(30%)	21(27%)	0.691
RSV infection	114(75%)	52(64%)	**0.060**
RV infection	39(28%)	21(28%)	0.922
RSV + RV coinfection vs. RSV single infection	25(22%)	14(27%)	0.500
RSV + RV coinfection vs. RV single infection	25(64%)	14(67%)	0.843
Recurrent wheezing	110(70%)	68(78%)	0.197
Asthma emergency visits past 12 months	26(17%)	23(27%)	**0.08**
Ever use of bronchodilator	105(69%)	71(82%)	**0.04**
Bronchodilator treatment past 12 months	47(31%)	44(52%)	**0.002**
Asthma maintenance treatment past 12 months	16(11%)	18(22%)	**0.020**
Admission for asthma	29(19%)	19(22%)	0.591
Ever wheezing	121(79%)	72(84%)	0.436
Wheezing in the past 12 months	49(32%)	41(48%)	**0.020**
Ever asthma	34(22%)	17(20%)	0.652
Wheezing after exercise	20(13%)	7(8%)	0.248
Dry cough at night	19(12%)	16(19%)	0.194

The unadjusted risk of current asthma at 4 years in infants with detectable nasal periostin was half that of children in whom periostin was undetected (OR: 0.522; CI 95% 0.303–0.899). In the multivariate analysis, adjusting for potential confounders (maternal atopy, parental asthma, atopic dermatitis, prematurity, RSV infection, and cigarette smoke exposure), the results showed a lower risk of asthma at 4 years of age in those infants with positive periostin detection at admission for bronchiolitis, independently of their atopic status (aOR: 0.511 CI 95% 0.284–0.918, p: 0.025) (Table 4).

## Discussion

Our results showed, for the first time, that infants admitted for bronchiolitis, who later developed asthma, with need of chronic treatment, by the age of 4, were significantly more likely to have nasal TSLP detection at admission. Indeed, regardless of their atopic status or the viral etiology, infants with nasal TSLP production were more likely to receive maintenance asthma treatment, including the combination of montelukast plus IGC, usually indicated for higher severity levels of asthma. Children with TSLP detection also tended to require more hospital admissions for recurrent wheezing. According to our data, and those obtained from experimental studies, TSLP seems to play a key role in asthma inception after respiratory viral infections. Han et al. [20] showed, in experimentally RSV-infected mice, that the administration of anti-TSLP antibodies before neonatal RSV infection significantly attenuated airway response to inhaled methacholine and reduced eosinophil numbers in the bronchoalveolar fluid. They postulated that neonatal RSV infection initiates a cascade that involves increased TSLP release from infected epithelial cells, which induces the upregulation of OX40 ligand (OX40L) expressed on lung dendritic cells, which may be critical for promoting the initial differentiation and expanding existence of Th2 cells and regulatory T cells (Tregs) [21]. This, in turn, initiates the polarization of RSV-specific T cells to a T2 phenotype so that re-exposure to RSV triggers the expansion of RSV-specific T2 memory cells and the enhanced development of airway hyperresponsiveness (AHR), accompanied by eosinophilic airway inflammation, mucus hyperproduction, and IL-13 release. More recently, Fan et al. [22] provided the first direct evidence that RSV non-structural protein (NS)1 breaks immune tolerance and induces airway inflammation and AHR in infected mice. Animal studies have also demonstrated that TSLP is necessary and sufficient for the development of T2 cytokine-associated airway inflammation, mediated through distinct immune cell cascades in the context of innate and adaptive T2 inflammation [23]. Salka et al. [24] recently reported that human infant airway epithelial cells respond to a virus mimic (double-stranded RNA) with robust production of TSLP, and in vivo, they also found that infants with higher TSLP nasal levels at admission for respiratory infections (not necessarily bronchiolitis), had an increased probability of respiratory hospitalizations or emergency room visits 12 months after discharge, suggesting a role of TSLP secretion during severe bronchiolitis in infancy and in asthma inception later in life. However, Chen et al. [25] did not find any association between several serum cytokine levels, including TSLP, in infants hospitalized for bronchiolitis and the frequency of recurrent wheezing episodes in a 2-year follow-up study. The authors explain their unexpected results to the fact that they tested serum samples rather than nasopharynx aspirates.

According to our results, the association between TSLP and asthma development seems to be independent of the atopic status. In line with our data, Vrsalovic et al. [26], recently described higher serum concentration of TSLP in asthmatics than in healthy children, but without any difference among the three different asthma phenotypes: allergic asthma, virus-induced asthma, and non-allergic asthma. Lin et al. [27] also reported higher levels of TSLP receptors in asthmatic patients than in healthy children, but similar concentrations between allergic and non-allergic asthmatic patients.

However, despite the strong association of TSLP with asthma, some studies have failed to find a consistent association between circulating TSLP and asthma development. The study conducted in the birth cohort from the Urban Environment and Childhood Asthma (URECA) found that the early presence of circulating TSLP was significantly associated with reduced incidence of recurrent wheeze in those children not sensitized to aeroallergen [28]. These differences could be explained by several factors. Firstly, there is a great deal of variation in the methodology among the different studies. Thus, while Chen et al [25] study included, as ours, only infants admitted with bronchiolitis, some authors [24] recruited infants less than 24 months hospitalized for a PCR-confirmed viral respiratory infection, regardless of whether it was bronchiolitis or a recurrent wheezing episode. Other studies recruited asthmatic children with a wide range of age [26, 27], whereas others followed up a cohort of newborns. In addition, most studies evaluate serum TSLP levels [25–28] that could be less reliable than nasal TSLP to express local TSLP production [24]. Also, genetic variation may have an impact beyond circulating TSLP protein expression, including on other genes or pathways as Biagnini Myers et al. [29] demonstrated. Murrison et al. [30] recently found that 90% of children with some defined TSLP risk genotypes and high nasal TSLP mRNA expression, had asthma compared with 40% of children without risk genotypes and with low nasal TSLP expression, finding no association between serum TSLP and asthma. These data suggest that childhood asthma may be modified by the combined effect of TLSP genotype and TSLP expression in the nasal epithelium and both factors should be taken into account when evaluating asthma risk.

In contrast to TSLP, the nasal detection of periostin at admission for bronchiolitis was associated, in our series, with a more favourable respiratory outcome. In fact, higher nasal periostin levels were significantly related to lower frequency of current asthma at age 4, ever diagnosis of asthma, maintenance asthma treatment prescription, and admissions for recurrent wheezing. Periostin is a distinct signature protein for the T2-high asthma phenotype in adults [31, 32] although its role in children is controversial. Some studies have found higher levels of serum periostin in children with asthma [19, 33–35] and some of them even reported a significant correlation between serum periostin levels and asthma severity [34]. However, other studies found similar serum periostin levels in children with severe asthma compared to those with controlled asthma [36, 37] or even lower periostin levels in children with severe uncontrolled asthma than in children with controlled asthma [38].

Regarding recurrent wheezing in preschool children, there are also inconclusive data about the association between serum periostin level and airway inflammation. Yooma et al. [39], in 2–5-year-old children, reported higher serum periostin levels in children with recurrent wheezing and in those who developed acute wheezing exacerbation in the subsequent year compared to healthy control children. Anderson et al. [40], in the Childhood Origins of Asthma (COAST) cohort study, demonstrated that, in children with atopic risk, a high periostin level at age 2 years was associated with a greater risk of asthma at age 6. However, a recent study by Guvenir et al. [41] evaluated the usefulness of serum periostin in wheezy preschool children for predicting the development of asthma in school ages, founding no difference in the levels of periostin between children with transient wheezing and children with asthma in both, preschool and school periods.

In relation to periostin detection in infants with acute bronchiolitis, our group, in a previous study, demonstrated for the first time that naturally occurring severe infections by the most common respiratory viruses, in hospitalized infants with bronchiolitis, induces nasal airway secretion of periostin when compared with healthy controls [12]. Regarding the association between periostin detection in infants with bronchiolitis and asthma development, Nanishi et al. [42] recently published a multicenter cohort study of infants with severe bronchiolitis, measured the serum periostin level at hospitalization, and grouped infants into three groups: low, intermediate, and high levels. They examined the association of periostin levels at entry with the development of asthma at 6 years of age. After adjusting for confounding factors, they found that, compared to the low periostin group, the asthma risk was significantly higher among infants in the intermediate group but non-significantly greater in the high-level group. After the stratified analysis, infants without IgE sensitization or parental asthma or eczema showed no significant periostin-outcome association, suggesting that high and moderate serum levels of periostin are associated with increased risk of asthma by age 6 years only among infants with severe bronchiolitis and allergic predisposition. The causal mediation analysis performed in that study demonstrated that there was no indirect (mediation) effect of periostin, suggesting that the effect of IgE sensitization on developing asthma was driven through pathways other than periostin. Our follow-up study, with a similar design to Nanishi’s et al [42], although conducted at a single center, with smaller sample size and with nasal rather than serum periostin detection, found, on the contrary, an inverse association between periostin detection and the development of asthma by age 4 years, independently of atopic risk factors such as atopic dermatitis or family history of asthma or atopy. Previous experimental studies found that periostin decreases allergic airway inflammation in mice. Gordon et al. [43] demonstrated that, compared with wild-type controls, periostin deficient mice developed increased AHR and serum IgE levels following allergen challenge. They speculated that periostin’s role in the airway is to act as a brake on allergen-induced IgE production and AHR. The mechanism of this effect could be explained by periostin’s regulation of the TGF-beta signaling pathway and the anti-inflammatory effects of TGF-beta-induced T regulatory cell differentiation. The study by Kondoh et al. [44] suggests that periostin strengthens the extracellular matrix structure of the intact alveolar wall and acts protectively during acute lung injury in mice. In view of the contradictory data and the limited evidence available, it seems that the role of periostin in the development of asthma in children with a history of severe bronchiolitis is far from being clarified.

Our study has several potential limitations. Only 27 infants had detectable nasal TSLP levels. We followed-up our patients up to the age of 4 years and as asthma can develop later in life, some of them could be misclassified now. That is why we have extended the follow-up until the age of 10 years.

## Conclusions

In summary, our results show, for the first time, a substantial positive correlation between TSLP detected in nasal secretions of infants with severe bronchiolitis and the likelihood of needing asthma maintenance treatment and respiratory admissions up to the age of 4 years. On the contrary, our analysis found a protective association between nasal periostin detection and current asthma at age 4 years, ever diagnosis of asthma, maintenance asthma treatment prescription, and admissions for recurrent wheezing. It is worth noting that ours is the second study to analyse the association between periostin in infants with bronchiolitis and the development of asthma later in life, and the results are contradictory. There is a need for further and more comprehensive studies on this subject with a similar methodology.

## Data Availability

The data that support the findings of this study are available from the corresponding author, upon reasonable request.

## Abbreviations

TSLP: Thymic stromal lymphopoietin
ISAAC: International Study of asthma and allergies in childhood
SABA: Short-acting beta2-agonists
OR: Odds ratio
aOR: Adjusted odds ratio
RSV: Respiratory syncytial virus
HRV: Rhinovirus
HMPV: Human metapneumovirus
HBoV: Human bocavirus
ADV: Adenovirus
PIV: Parainfluenza virus
ILC2: Innate lymphoid cells
ICU: Intensive care unit
NPA: Nasopharyngeal
ISCIII: Instituto de Salud Carlos III
RT-PCR: Reverse transcription-polymerase chain reaction
ELISA: Enzyme linked immunosorbent assay
ANOVA: Analysis of variance
Tregs: Regulatory T cells
AHR: Airway hyperresponsiveness
NS protein: Non-structural protein
URECA: Urban Environment and Childhood Asthma
COAST: Childhood Origins of Asthma
TGF-beta: Transforming growth factor beta
